# *Meloidogyne incognita*-Induced Giant Cells in Tomato and the Impact of Acetic Acid

**DOI:** 10.3390/plants14071015

**Published:** 2025-03-24

**Authors:** Christianna Meidani, Konstantinos Telioglanidis, Eleni Giannoutsou, Nikoleta Ntalli, Ioannis Dimosthenis S. Adamakis

**Affiliations:** 1 Section of Botany, Department of Biology, National and Kapodistrian University of Athens, 15784 Athens, Greece; sbi1200150@biol.uoa.gr (C.M.); konstantinostelioglanidis@biol.uoa.gr (K.T.); egianno@biol.uoa.gr (E.G.); iadamaki@biol.uoa.gr (I.D.S.A.); 2 Analytical Chemistry and Pesticides Laboratory, Department of Agriculture Crop Production and Rural Environment, University of Thessaly, 38446 Volos, Greece

**Keywords:** acetic acid, cell wall, giant cells, extensins, *Meloidogyne incognita*, tomato

## Abstract

The plant parasitic root-knot nematodes of the species *Meloidogyne incognita* infect many cultivated plants, one of which is the tomato (*Solanum lycopersicum)*. To be fed, *M. incognita* selects unique feeding sites inside the root and induces the formation of large galls (knots) encompassing the so-called giant cells (GCs). In the present study, a comparative analysis of the GCs/root cell and cell wall components between *M. incognita*-infected and uninfected tomato plants and plants pre-treated with the plant biostimulant and nematicide acetic acid (AA) was carried out. Pectin, hemicellulose and extensin epitopes were detected in tomato root sections. *M. incognita*-induced GCs in tomato roots had cell walls with arabinans, unesterified/methylesterified homogalacturonans and xyloglucans, but were devoid of mannans and extensins. Interestingly, the above epitope distribution also differed in root sections made near the formed root knot, proximal to the root cap. Moreover, it seemed that AA was able to induce the deposition of extensins in AA-treated, *M. incognita*-uninfected roots and hamper the GC development in AA-treated, *M. incognita*-infected roots. According to the above the AA, stimulates natural defense mechanisms in tomato, thus protecting it from nematode infestation.

## 1. Introduction

Root-knot nematodes (RKNs; *Meloidogyne* spp.) are devastating plant parasites that severely impact agricultural productivity by infesting the roots of various cultivated crops. Tomatoes (*Solanum lycopersicum* L.), one of the world’s most economically significant crops, are particularly vulnerable to these pests [1]. Within the *Meloidogyne* genus, *Meloidogyne incognita* stands out as a significant concern due to its widespread occurrence and the substantial yield losses it causes in both commercial and subsistence farming systems [2]. These nematodes have a complex life cycle, with second-stage juveniles (J2) serving as the primary infective stage. Upon entering the host plant root, J2 nematodes migrate through the root tissues towards the vascular cylinder and manipulate the host cells by inducing the formation of specialized feeding structures known as giant cells (GCs) [3]. This process involves the transformation of four to eight parenchyma cells into multinucleate, metabolically active cells that support the nematode’s development. The resulting feeding site is encapsulated within a macroscopically visible gall, or “knot,” which is the hallmark of RKN infestation [4].

A critical aspect of RKN infection is the secretion of effector molecules by the nematodes during root invasion. These effectors enable the nematodes to breach root defenses, navigate through tissues, and establish feeding sites. This interaction is facilitated by significant modifications in the host cell wall composition, which play a pivotal role in the plant’s defense response [5,6]. During GC development, the cell walls undergo marked remodeling, characterized by the accumulation of highly methyl-esterified homogalacturonans (HGs), xyloglucans, and arabinans. These changes provide the necessary flexibility and structural support to accommodate the substantial increase in cell volume associated with GC maturation [6,7].

Given the increasing restrictions on synthetic nematicides due to environmental and health concerns [8,9], there is a pressing need for sustainable alternatives. Plant-based compounds, particularly extracts from Lamiaceae species and other botanical sources, have shown promise as eco-friendly nematicides [10]. One such compound is acetic acid (AA), a carboxylic acid naturally occurring as a secondary metabolite in the fruits of *Melia azedarach* [11], which may offer potential for nematode control. Acetic acid has demonstrated potent nematicidal activity, including the ability to paralyze or kill *M. incognita* J2, disrupt their body turgidity, and induce morphological changes [12]. Beyond its direct nematicidal effects, AA has also been shown to inhibit egg hatching and reduce the release of infective J2 individuals in related species like *M. javanica* [13]. Furthermore, AA exhibits plant biostimulant properties, enhancing plant resilience to abiotic stresses and potentially bolstering innate defense mechanisms [14,15].

Acetic acid (AA) offers a dual advantage by potentially disrupting the nematode life cycle while enhancing plant defense mechanisms. However, its specific impact on the structural properties of cell walls during *Meloidogyne incognita* infection is not well understood. We hypothesize that AA alters key cell wall components, thereby impairing giant cell formation and reducing nematode infection in tomato plants. This study aims to analyze the cell wall composition of both infected and uninfected tomato roots following AA treatment, with a focus on components involved in giant cell formation, to assess AA’s potential as a sustainable, eco-friendly nematicide and its role in integrated pest management.

## 2. Results

**Figure 1:**
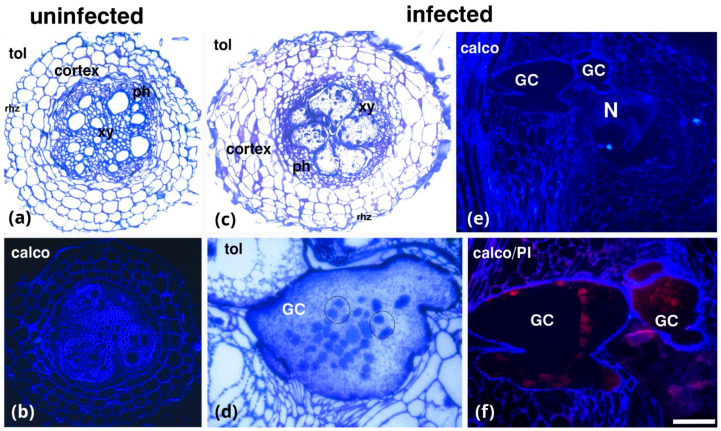
Comparative anatomy of uninfected (**a**,**b**) and infected (**c**–**f**) (25 dpi) tomato roots. Either transverse sections (**a**–**c**) or longitudinal sections (**d**–**f**) are depicted, stained with toluidine blue (**a**,**c**,**d**), calcofluor-white (**b**,**e**), or with a combination of calcofluor-white and propidium iodide (**f**). Giant cells (**c**–**f**) show their typical characteristics, e.g., numerous nuclei (circles in (**d**)). GCs, giant cells; Rhz, rhizodermis; ph, phloem; xy, xylem elements; N, nematode; tol, toluidine blue; calco, calcofluor-white; propidium iodine, PI. Scale bar: 50 µm.

Comparing uninfected roots stained with toluidine blue (Figure 1a) and calcofluor-white (Figure 1b) to infected roots stained with either toluidine blue (Figure 1c,d) or calcofluor-white (Figure 1e,f) in transverse (Figure 1c,d) and longitudinal (Figure 1e,f) sections allowed for the observation of roots’ anatomical features. This analysis confirmed that *M. incognita* infection induced the formation of giant cells (GCs) in tomato roots (Figure 1c–e). The number of GCs varied across different replicates (3–6) (Supplementary Figure S1), and in some cases, the nematode was visible inside the root knot (Supplementary Figure S1a–c). Additionally, the diameter of GCs ranged from 40 to 60 μm. At the central vascular cylinder of uninfected roots, typical features of development such as the xylem vessels and phloem elements were observed, while at the root periphery, cortex cells were observed (Figure 1a). Xylem vessels were more intensely stained by calcofluor-white (Figure 1b). The GCs located in the vascular cylinder of infected plants were in close contact with the nematode (Figure 1e), highly vacuolated, and contained multiple nuclei (Figure 1d,f)—a feature also confirmed by propidium iodide staining (Figure 1f). The GC’s wall was thickened and intensively stained by calcofluor-white (Figure 1f).

**Figure 2:**
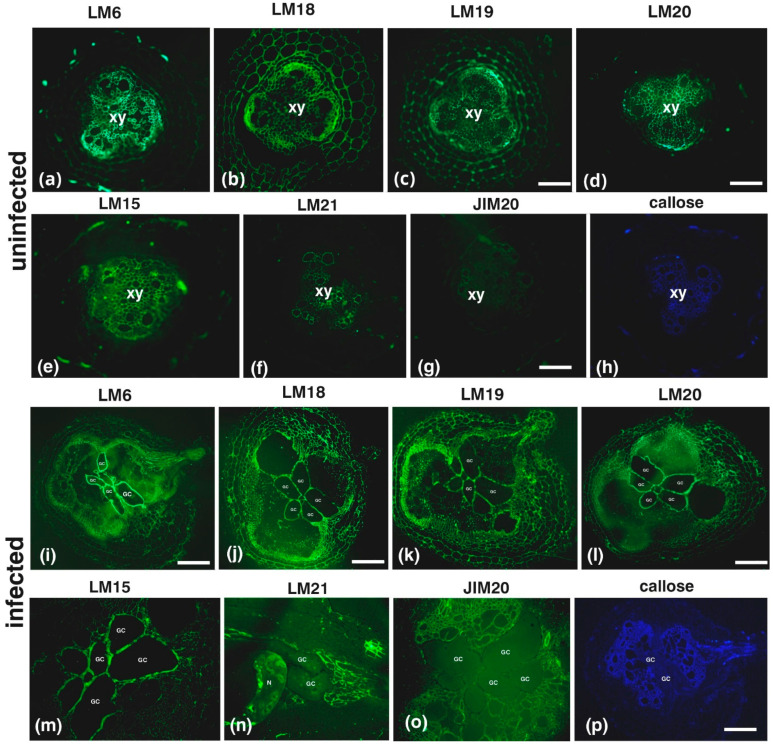
Distribution of cell wall components in uninfected tomato roots (**a**–**h**) and infected tomato roots (**i**–**p**). In uninfected roots, pectic arabinans (LM6) (**a**) were primarily detected in xylem vessels. Partially methylesterified HGs (LM18) (**b**) and demethylesterified HGs (LM19) (**c**) were mainly localized in phloem elements. Methylesterified HGs (LM20) (**d**) were present in both xylem vessels and phloem. Xyloglucan (LM15) (**e**) and mannan (LM21) (**f**) epitopes were detected in xylem vessels. Extensin epitopes (JIM20) (**g**) were absent and callose staining (aniline blue) was weak (**h**). In infected tomato roots, giant cell (GC) walls were enriched in pectic arabinans (LM6), HGs with various degrees of methylesterification (LM18–LM20) (**i**–**l**), and xyloglucans (LM15) (**m**). Mannan (LM21) (**n**), extensins (JIM20) (**o**), and aniline blue staining (**p**) were absent in GCs. xy; xylem elements; N: nematode. Scale bars: 50 µm.

**Figure 3:**
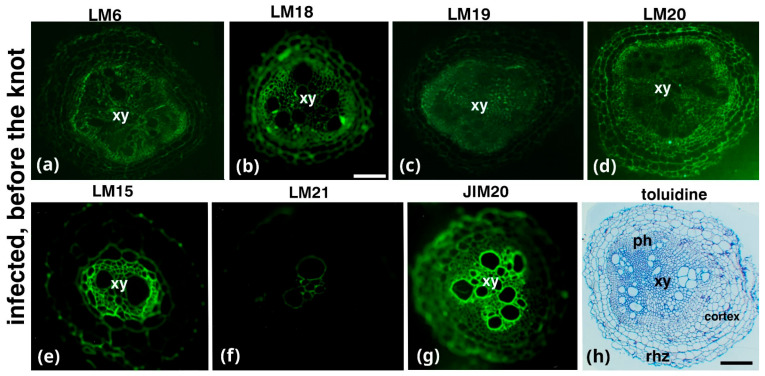
Immunolabeling (**a**–**g**) and histological (**h**) analysis of root sections from a region near the knot and distal to the root cap (**a**,**c**,**e**,**g**). Immunolabeling for pectic arabinans (LM6), demethylesterified HGs (LM19), xyloglucans (LM15), and extensins (JIM20) was observed in the xylem vessels of the central cylinder. (**b**,**d**) Partially methylesterified HGs (LM18) and methylesterified HGs (LM20) show intense signals in the phloem. (**h**) Toluidine blue staining reveals deformation of the central cylinder. Rhz, rhizodermis; ph, phloem; xy, xylem elements. Scale bars: 50 µm.

Callose staining and hemicellulose, pectin and extensin epitope immunolabeling was conducted on root sections of uninfected plants (Figure 2a–h) or infected plants in the root knot (Figure 2i–p) and in a root area near the knot (the images were taken distal to the root cap, approximately 3 mm anterior to the root gall) (Figure 3). In uninfected roots, pectic arabinans (LM6; Figure 2a) were mostly present in the xylem vessels, while partially methylesterified HGs (LM18; Figure 2b) and demethylesterified HGs (LM19; Figure 2c) were mostly found in the phloem elements. Methylesterified HGs (LM20; Figure 2d) were also found in the xylem vessels along with xyloglucan (LM15; Figure 2e) and mannan epitopes (LM21; Figure 2f). Uninfected root sections were devoid of extensin epitopes (JIM20; Figure 2g) and had a very weak aniline blue signal (callose; Figure 2h). The cell walls of tomato GCs were enriched with pectic epitopes (in particular arabinans (Figure 2i) and HGs (with various degrees of methylesterification (Figure 2j–l)) and xyloglucans (Figure 2m), but were lacking mannan (Figure 2n), extensins (Figure 2o), and aniline blue (Figure 2p) signals.

Root sections from a root area near the knot, which is distal to the root cap, exhibited relatively low LM6 (arabinans) and LM19 (demethylesterified HGs) and intense LM15 (xyloglucans) and JIM20 (extensin) signals (Figure 3a,c,e,g, respectively) in the xylem vessels of the central cylinder, and an intense signal of the LM18 and LM20 epitopes in the phloem (Figure 3b,d, respectively). Toluidine blue staining revealed central-cylinder deformation (Figure 3h).

**Figure 4:**
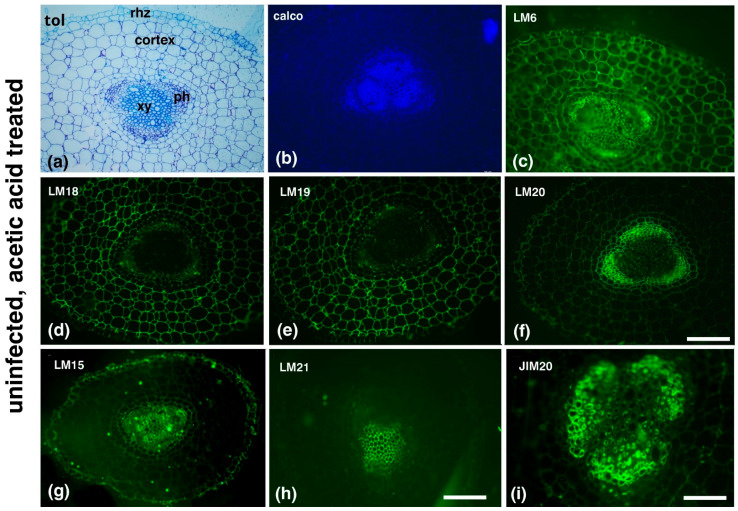
Effects of acetic acid treatment (AA) treatment in uninfected roots. AA treatment in uninfected roots did not result in anatomical deformations, as shown by toluidine blue (**a**) and calcofluor-white (**b**) staining. LM6 (arabinans) shows a broader distribution (**c**), while partially methylesterified HGs (LM18) (**d**) and demethylesterified HGs (LM19) (**e**) are absent from the central cylinder. In contrast, LM20 (methylesterified HGs) is predominantly observed in the phloem elements (**f**). LM15 (xyloglucans) was present in the xylem vessels and the rhizodermis (**g**), LM21 (mannans) was found in the xylem (**h**), and JIM20 (extensins) was intensely prominent in the phloem (**i**). Rhz, rhizodermis; ph, phloem; xy, xylem elements; tol, toluidine blue; calco, calcofluor-white. Scale bars: 50 µm.

AA treatment in uninfected roots (Figure 4) did not cause any anatomical deformations, as revealed by the toluidine blue (Figure 4a) and calcofluor-white (Figure 4b) staining, but it affected the distribution of the pectic epitopes (Figure 4c–f) when compared to the AA-untreated, uninfected plants (see Figure 2a–d). In particular, LM6 (arabinans) was more broadly distributed (Figure 4c), whilst partially methylesterified HGs (LM18) (b) and demethylesterified HGs (LM19) were scarce in the central cylinder and more prominent in the cortex (Figure 4d,e, respectively). Meanwhile, LM20 (methylesterified HGs) was observed mostly in the phloem elements (Figure 4f) and displayed a low signal in the cortex. LM15 (xyloglucans) was present in the xylem vessels but also in the rhizodermis (Figure 4g); LM21 (mannans) was present in the xylem (Figure 4h), while JIM20 (extensins) was present in the phloem (Figure 4i).

**Figure 5:**
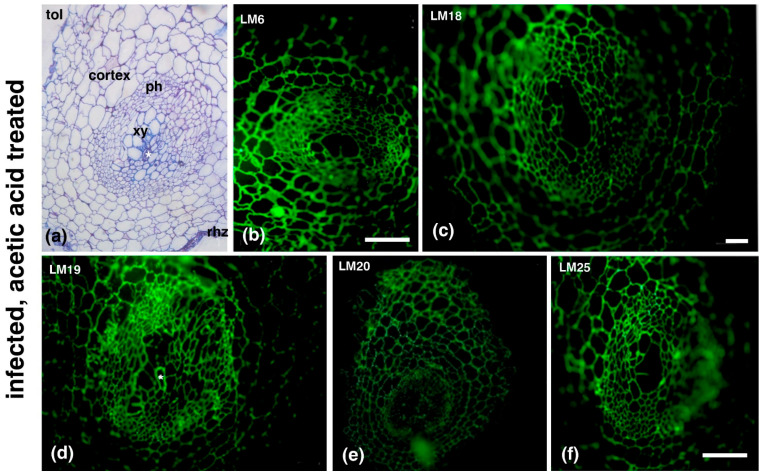
Effects of acetic acid treatment (AA) treatment on giant cell (GC) formation and cell wall composition in *M. incognita*-infected tomato roots. AA treatment altered GC development in infected plants, resulting in underdeveloped GCs lacking typical characteristics (asterisk in (**a**,**d**)). Additionally, AA-treated, infected GCs exhibited changes in cell wall epitope distribution, with an absence of LM20-HG (**e**) and an enrichment of LM6, LM18, LM19, and LM25-HG epitopes ((**b**,**c**,**d**,**f**), respectively). Rhz, rhizodermis; ph, phloem; xy, xylem elements; tol, toluidine blue. Scale bars: 50 µm.

AA treatment affected GC formation (Figure 5) in *M. incognita*-infected plants. GCs in AA-treated, infected plants appeared underdeveloped and lacked the typical characteristics of GCs (see Figure 5a and Figure 1c). Moreover, in the examined replicates, no more than one GC per root knot was ever detected, and AA-treated GCs appeared relatively small, with a diameter of approximately 20 μm (Supplementary Figure S2). The epitope distribution of AA-treated, infected plant GCs was also altered, with their walls devoid of methylesterified HGs, as detected by LM20 antibody (Figure 5e), but rich in demethylesterified HGs, as detected by LM18 and LM19 antibodies (Figure 5c,d, respectively). Furthermore, the infected-plant GCs’ walls were also rich in arabinans and xyloglucans, as detected by LM6 and LM25 antibody respectively (Figure 5b and 5f).

## 3. Discussion

The formation of giant cells (GCs) in *Solanum lycopersicum* (tomato) in response to *Meloidogyne incognita* infestation has been documented since the 1960s–1970s [16,17], but the exact mechanism governing their formation remains poorly understood. While studies on GC wall composition have been conducted in other plant species, such as *Zea mays* (maize) [6], *Abelmoschus esculentus* (okra) [18], *Vigna angularis* (Azuki bean) [6], and the model plant *Arabidopsis thaliana* [6,7], data specific to tomato are limited. Our findings reveal strong staining for pectic epitopes, particularly arabinans (LM6) and partially demethylesterified homogalacturonans (HGs) (LM19), in tomato GCs (Figure 2). These components, absent in Azuki bean [6], *Arabidopsis* [6,7], and maize [6], appear more prominently in tomato, suggesting a distinct host response to nematode infestation. The observed alterations in pectin composition resemble plant responses to other pests such as aphids [19], aligning with previous studies highlighting how nematodes manipulate cell walls to optimize nutrient uptake. In *Arabidopsis*, *M. javanica* infection induces the expression of pectin methylesterases in knot structures, facilitating the accessibility to host pectate lyases and polygalacturonases, thereby enhancing the formation of nematode feeding sites [20,21].

The extensive pectic modifications observed suggest that nematodes actively reshape the host cell wall to establish these sites. Notably, this remodeling follows a distinct pattern in tomato, potentially reflecting the nematode’s adaptation to specific host structural traits [22]. The methylesterification status of homogalacturonan (HG) plays a pivotal role in plant immunity by influencing cell wall porosity, elasticity, and expansion capacity. Pectins are central regulators of plant–pathogen interactions, with plants modulating pectin methylation and substitution patterns to counter colonization [23,24,25]. Specifically, HG demethylesterification promotes the formation of Ca^2^⁺-mediated egg-box complexes, reinforcing cell walls and enhancing resistance [26,27]. Additionally, altered HG methylesterification can trigger the release of signaling molecules, such as oligogalacturonides or methanol, which elicit robust defense responses [28].

Arabinan likely contributes to plant defense by reinforcing cell wall adhesion and mechanical properties, though its precise role remains unclear due to the absence of mutants exclusively deficient in arabinan without affecting other arabinose-containing polymers. Some arabinan-deficient mutants exhibit increased susceptibility to fungal pathogens, suggesting a synergistic role with pectin methylesterases in defense [29]. However, our findings challenge this notion, as an increased arabinan signal was observed in tomato giant cells (GCs), indicating a potentially distinct role in nematode-induced cell wall modifications. Arabinans may be present either in arabinogalactan proteins (AGPs) or as part of side chains of rhamnogalacturonans-I pectin-derived oligogalacturonides. It is possible that arabinans present in pectic residues favor cell wall elasticity, allowing for the formation of a successful nematode feeding site.

Despite variations in cell wall composition across plant species, a core set of polysaccharides, such as xyloglucans (LM15), consistently appear in GCs. This suggests that GC formation relies on the modulation of pre-existing cell wall components. These findings support the hypothesis that *Meloidogyne incognita* has evolved mechanisms to exploit host cell wall structures for successful parasitism [30]. The nematode’s ability to recognize host-specific features and modify cell walls underscores the intricate interplay between host defenses and nematode adaptation.

Extensins, known for their role in plant defense, contribute to cell wall reinforcement and enhance resistance to pathogen attacks [31,32,33]. Extensin expression in plant cell walls is triggered by pathogenic infections, biological elicitors, and physical damage, with fungal and bacterial infections being the most common inducers. Extensins strengthen plant defense by forming physical barriers, binding to pathogen surfaces, and accumulating at infection sites to restrict pathogen spread [34,35]. Similarly, nematode infestations, such as those caused by *Meloidogyne* and *Globodera* species, also induce extensin gene expression at feeding sites, suggesting a role in plant–nematode interactions [36,37]. This aligns with previous findings in *Nicotiana tabacum*, where extensin gene expression is strongly induced following *Meloidogyne javanica* infection [37]. Our findings support these observations, showing that extensin accumulates in root areas adjacent to the infection (Figure 3). This may indicate a defensive response aimed at protecting surrounding root tissues from nematode invasion. These results underscore the multifaceted role of extensins in plant defense, functioning as both structural reinforcements and integral components of broader immune responses.

This study explores a novel plant defense strategy by examining the effects of acetic acid (AA) on nematode-induced giant cell (GC) formation. A previous study on *Solanum lycopersicum* plants infected with *Meloidogyne javanica* and treated with the same AA concentration used in this research reported a significant reduction in gall formation—60 galls per gram of root compared to 100 galls per gram in the controls [13]. The researchers concluded that AA likely disrupts the establishment of *Meloidogyne* feeding sites. In our study, AA-treated, infected tomato plants lacked the characteristic GC morphology, suggesting that beyond its known nematicidal properties [13,38], AA alters GC structure in a way that impairs nematode feeding site formation. Although AA treatment did not completely prevent nematode infection, it significantly inhibited GC formation, a crucial factor for nematode feeding.

Interestingly, even in uninfected, AA-treated plants, arabinans and extensins were consistently detected in root tissues (Figure 4), both of which contribute to cell wall reinforcement, a key mechanism in pathogen defense [29,33]. This suggests that AA enhances resistance not only to nematodes but also to other pathogens and environmental stresses. These findings highlight the dual role of AA—reducing *M. incognita* infection while simultaneously strengthening plant tolerance to abiotic challenges [39,40].

Beyond its role in nematode suppression, AA functions as a biostimulant, enhancing plant resilience to abiotic stresses such as drought and salinity [41,42]. Resistance inducers like AA have emerged as promising tools in plant protection, offering environmentally friendly disease management strategies while providing insights into induced resistance mechanisms [43]. Specifically, AA appears to act as a priming agent affecting cell wall composition [44]—an aspect already established for other inducers such as hexanoic acid [45], frass [46], salicylic acid, and methyl jasmonate [47]. These compounds commonly trigger cell wall fortification through callose or lignin deposition, key elements of plant resistance.

In conclusion, this research highlights the complex interplay between nematodes and their host plants, emphasizing the role of cell wall modulation in the establishment of feeding sites. The unique composition of tomato GCs, along with the promising results of acetic acid treatment, sheds light on new strategies for managing nematode infestations and improving plant health. Further studies are needed to explore the precise mechanisms through which acetic acid influences cell wall composition and to assess its potential as a biostimulant for broader agricultural applications.

## 4. Materials and Methods

### 4.1. Nematode Rearing and Collection

A *Meloidogyne incognita* population of Greek origin [13] was reared on 7 week-old tomato plants (*Solanum lycopersicum* Mill. cv. Belladonna), a cultivar known for its high susceptibility to root-knot nematode (RKN) infestation. Thirty plants were cultivated in a growth chamber under controlled conditions of 25 ± 1 °C, 60% relative humidity (RH), and a 16 h light/8 h dark photoperiod. Illumination was provided by white fluorescent lamps at an intensity of 350 μmol m^−2^ s^−1^. Watering was carried out every three days, with no extra fertilization. The plants were maintained in separate trays and grown in nematode-free soil, collected from Aristotle University experimental farm, as described below, using 30 cm pots. Sixty days after artificial inoculation, roots were collected for nematode extraction. Second-stage juveniles (J2), extracted from 60-day nematode-infested roots according to a modified method from Hussey and Barker [48], were used for the bioassays. In short, eggs were isolated using sodium hypochlorite. Second-stage juveniles (J2) were hatched in modified Baermann funnels at 28 °C. Second-stage juveniles (J2) that hatched within the first three days were discarded, while those collected during the following 24 h were used for the experiments.

### 4.2. Plant Infestation, Acetic Acid Treatments, and Root Chemical Fixation

Nematode-free soil was collected from the experimental farm of the School of Agriculture, Aristotle University of Thessaloniki, and it was characterized as a clay loam with 1.3% organic matter and a pH of 7.8. It was divided into four equal parts (1 kg/part), which represented the experimental treatments (uninfected; infected; uninfected, AA-treated; infected, AA-treated). The first part of the soil was artificially inoculated with a suspension of 2500 J2, mixed by stirring for a better distribution of nematodes and left in the dark for 24 h. Then, it was sieved again through a 3 mm pore size sieve and treated with acetic acid (AA) at a concentration of 650 mg/kg [13] (infected, AA-treated). After the fortification of the soil with the acetic acid, the soil was stirred and passed through the 3 mm pores two times to reassure the homogeneous distribution. The second part of the soil only accepted the treatment of acetic acid (uninfected, AA-treated) with no addition of J2s. The third part of the soil was treated with water and nematode suspension and used to witness the activity of nematodes alone (infected). Finally, the fourth part was treated with distilled water (uninfected).

Following the pot experiment described in [13], each experimental treatment was conducted in two replicates, with five plants per replicate (10 plants in total per experimental treatment). The soil for each treatment (1 kg) was divided into five pots, each containing 200 g of soil, and used to transplant a six-leaf-stage tomato plant (*Solanum lycopersicum* Mill. cv. Belladonna). The plants were maintained in a growth chamber under a constant 16 h light/8 h dark photoperiod at an ambient temperature of 25 ± 1 °C, with white fluorescent lamps providing a light intensity of 120 μmol m^−2^·s^−1^ and 60% relative humidity (RH). As before, plants were placed in separate trays, and 5 cm diameter pots were used. Watering was carried out every three days, with no extra fertilization.

Twenty five days post-infection (25 dpi), root segments from all treatments were chemically fixed and embedded in LRW (London Resin White) as described in Meidani et al. (2019, 2021) [7,49]. In short, root tips were fixed in 2% paraformaldehyde in PEM buffer (50 mM PIPES, 5 mM EGTA, 5 mM MgSO_4_, pH 6.8) with 0.1% glutaraldehyde at 4 °C for 1.5 h. After washing with PEM, the samples were dehydrated on ice using a graded ethanol series (10–90% in 10% increments, followed by absolute ethanol). At the 30% step, 0.25% OsO_4_ was added for post-fixation. The specimens were then infiltrated with London Resin White diluted in ethanol (10–90% in successive 1 h steps at 4 °C), followed by overnight infiltration with pure resin, embedding in plastic capsules, and polymerization at 60 °C for 48 h. All chemicals and reagents were purchased from Applichem (Darmstadt, Germany), Merck (Darmstadt, Germany), PolySciences (Niles, IL, USA), SERVA (Heidelberg, Germany) and Sigma-Aldrich (Taufkirchen, Germany), unless otherwise stated. We sectioned at least five different knots/root segments from each plant, ensuring the full range of variation. Root sections were prepared for cell wall epitope immunofluorescence as described in Giannoutsou et al. (2020) [50]. The antibodies used are shown in Table 1. Sections were also stained with calcofluor-white (0.01% (*w*/*v*) in PBS buffer), propidium iodide (0.1 mg/mL in phosphate-buffered saline), and aniline blue (0.05% (*w*/*v*) in 0.07 M K_2_HPO_4_ buffer, pH 8.5) for cellulose, nuclei, and callose visualization, respectively. Also, staining with 0.5% (*w*/*v*) toluidine blue O in a 1% (*w*/*v*) borax aqueous solution was conducted [7]. The figures obtained depict representative cases reflecting typical morphological and structural characteristics.

## Figures and Tables

**Table 1:** List of primary monoclonal antibodies used in the current study for the immunolocalization of different cell wall epitopes. All descriptions and/or references for the antibodies and their use can be found at www.kerafast.com (accessed on 15 February 2025) and www.plantprobes.net (accessed on 15 February 2025).

Antibody	Epitope Recognized
	*Hemicelluloses*
LM15/LM25	XXXG/XXLG and XLLG motifs of xyloglucan
LM21	β-(1–4)-manno-oligosaccharides from DP2 to DP5
	*Pectins*
LM6	(1→5)-α-L-arabinans
LM18	Homogalacturonan (HG) domain of pectic polysaccharides (binds to both partially methyl-esterified and unesterified HGs)
LM19	HG domain of pectic polysaccharides (binds strongly to unesterified HGs)
LM20	HG domain of pectic polysaccharides (requires methyl-esters for HG recognition, does not bind to unesterified HGs)
	*Extensins (EXTs)*
JIM20	Extensin glucoprotein

## Data Availability

Data are contained within the article and Supplementary Materials.

## Supplementary Materials

The following supporting information can be downloaded at: https://www.mdpi.com/article/10.3390/plants14071015/s1, Figure S1. Variations in root knot Giant Cell (GC) morphology among different replicates. Cross-sectioned toluidine blue-stained root knots, with the nematode visible in some cases (a). GCs: Giant Cells; N: Nematode. Scale bar: 50 µm. Figure S2. Acetic acid-treated root knots across different replicates. Cross-sectioned root knots under DIC optics, showing only one GC visible per replicate. GCs: Giant Cells. Scale bar: 50 µm.
