# Supplementary material for: Meloidogyne incognita-Induced Giant Cells in Tomato and the Impact of Acetic Acid
Source: Plants (Basel). 2025 Mar 24;14(7):1015. doi: 10.3390/plants14071015 (PMC11990625; doi:10.3390/plants14071015)
Supplement: Supplementary file 1 [file plants-14-01015-s001.zip › plants-3521985-supplementary.pdf]

Meloidogyne incognita-Induced Giant Cells in Tomato and the Impact of Acetic Acid

Christianna Meidani [1], Konstantinos Telioglanidis [1], Eleni Giannoutsou [1], Nikoleta Ntalli [2,*] and Ioannis Dimosthenis S. Adamakis [1]

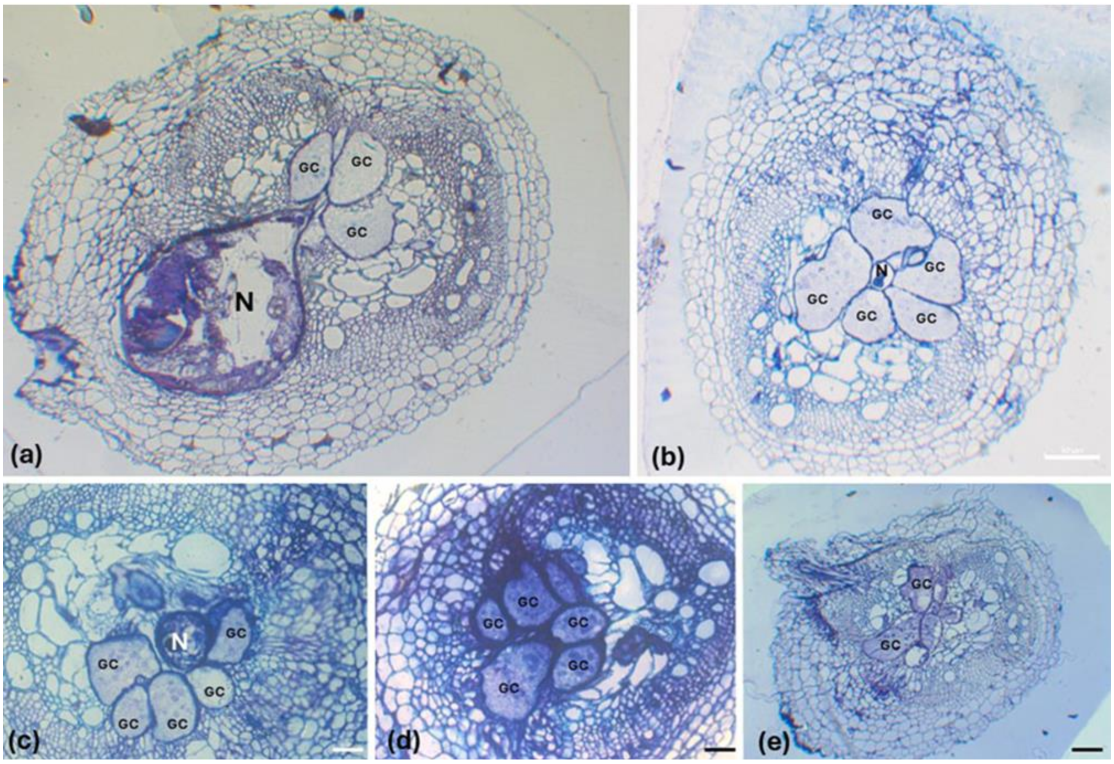

Figure S1. Variations in root knot Giant Cell (GC) morphology among different replicates. Cross-sectioned toluidine blue-stained root knots, with the nematode visible in some cases (a, b, c). GCs: Giant Cells; N: Nematode. Scale bar: 50 μm.

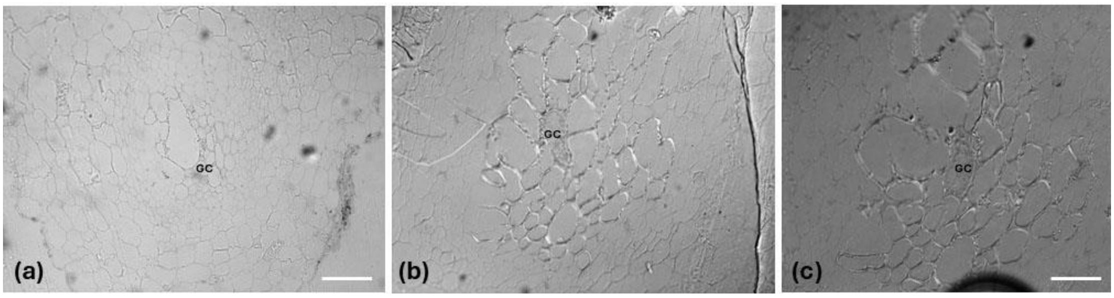

Figure S2. Acetic acid-treated root knots across different replicates. Cross-sectioned root knots under DIC optics, showing only one GC visible per replicate. GCs: Giant Cells. Scale bar: 50 μm.
